# The Use of a Hybrid Pillar and Its Importance for Aesthetic Rehabilitation and Tissue Stability: A Clinical Report

**DOI:** 10.1155/2018/6850720

**Published:** 2018-07-02

**Authors:** Guilherme da Gama Ramos, Danilo Lazzari Ciotti, Samuel Rehder Wimmers Ferreira, Maide Rehder Wimmers Ferreira Margarido, Raquel Adriano Dantas, Marina Nottingham Guerreiro

**Affiliations:** ^1^São Leopoldo Mandic Institute and Research Center, R. José Rocha Junqueira 13, 13045-755 Campinas, SP, Brazil; ^2^Paulista Association of Dental Surgeons, Rua José Nardon 177, 13419-000 Piracicaba, SP, Brazil

## Abstract

In the past, aesthetics had a secondary role in implant rehabilitation. Nowadays, the search for a perfect and harmonious aesthetic has stimulated the development of new materials and techniques. Due to this aesthetic requirement, the hybrid abutment (titanium link + zirconia) emerged as an alternative to metallic pillars. The hybrid abutment made a more favorable aesthetic possible, provided reliable mechanical properties, and increased biocompatibility to the surrounding tissues. Additionally, the individual zirconia abutment improves the emergency profile and the final white aesthetics. The objective of this paper is to report a clinical case with a manufactured individualized hybrid abutment for a metal-free indirect restoration, showing the applicability, mechanical properties, and biocompatibility of the hybrid abutment.

## 1. Introduction

Aesthetic demands led us to new concepts and prosthetic resources in dentistry. We now use new materials with optical, mechanical, and biological properties [1]. Zirconia abutment fabrication and metal-free-implant-supported prostheses favor a better aesthetic situation than the metal counterpart [2]. These methods allow translucency in dental restoration, provide gingival tissue shade reduction, and result in a very natural and healthy appearance [3].

Zirconia abutments not only allow light transmission in the same manner as natural teeth [2, 4, 5] but also present reliable mechanical properties and soft tissue biocompatibility.

The aesthetic success of implant-supported prostheses is strongly related to the surrounding soft tissue appearance. Unlike metal abutments, which cause an unpleasant appearance in a fine gingival biotype [6], the use of zirconia abutment allows light scattering and customization for each individual case. This creates an emergence profile that provides color, shape, and gingival symmetry similar to natural teeth [4, 5].

As observed in several studies, zirconia has shown satisfactory results in aesthetic of prosthetic crowns as well as in adjacent gingival tissues [2, 7–11]. Due to the increase of the use of aesthetic abutments and restorations, new technologies have appeared, such as CAD/CAM systems [12, 13].

The CAD/CAM system consists of a planning and production computerized system for crowns, facets, inlays, onlays, crown copings, implant abutments, and even zirconia structures for fixed and removable partial prostheses. Through this system, pieces are fabricated with quality, high accuracy, minimal human intervention, error reduction during production, and lowered manufacturing costs [13, 14].

The objective of this paper was to show the importance of aesthetic customized prosthetic abutment and its indications and advantages and disadvantages, along with a clinical case presentation.

## 2. Case Presentation

A 46-year-old male patient with absence of element 24 presents with a need for aesthetic rehabilitation. The patient had tooth extraction indicated due to root fracture. After Anthogyr PX 4.0 × 8 mm implant installation, a provisional restauration for gingival tissue maintenance was made, in respect of the ideal critical and subcritical contour, providing a more predictable and stable gingival emergence profile.

**Figure 1:**
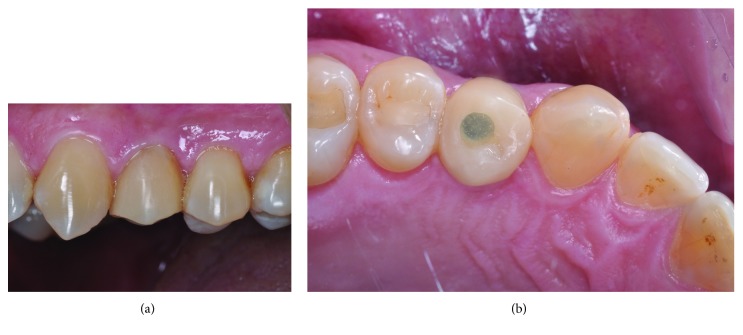
Provisional restauration properly prepared and screwed on the implant.

During the osseointegration period (120 days), the temporary customized crown did not have any occlusal contact. After this period, the acrylic temporary crown, previously prepared, was adjusted. For a better gingival tissue conditioning, we proceeded with temporary crown reassembly. Figures 1(a) and 1(b) display the temporary component properly prepared and screwed on the implant.

**Figure 2:**
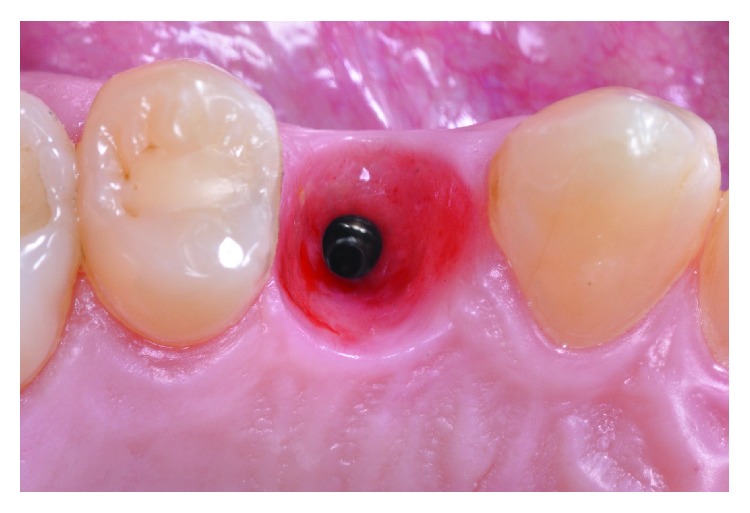
Gingival contour and emergence profile obtained by the provisional restauration.

Figure 2 shows an excellent emergence profile and the quality of the soft tissue obtained by the provisional component that was made in respect of the gingival biotype, and a concave critical and subcritical transmucosal emergence profile ensured the soft tissue quality [15#x2013;^17^].

**Figure 3:**
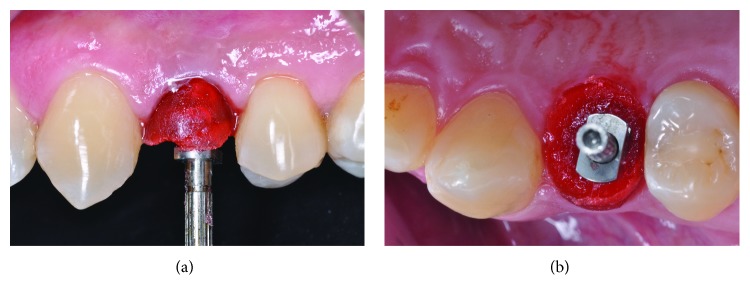
Molding with customized transfer in position, made with Pattern Resin.

**Figure 4:**
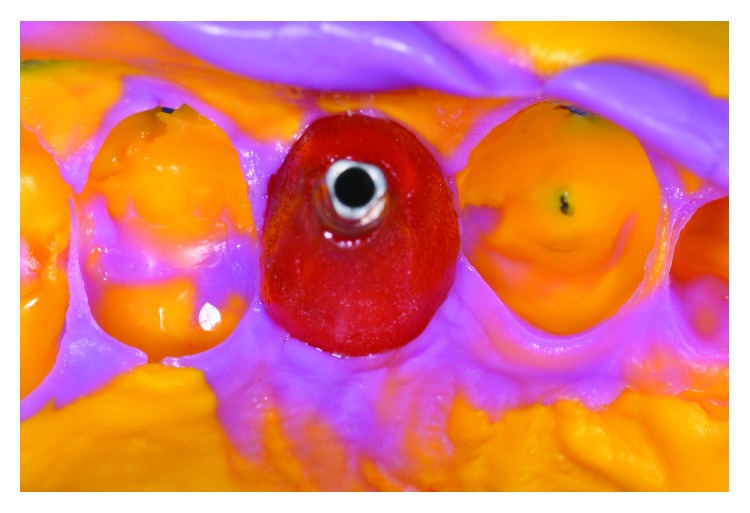
Transfer mold.

For the preparation of the working cast, customized transfer was used (Figures 3(a) and 3(b)) and molding was done with polyvinyl siloxane (Figure 4).

**Figure 5:**
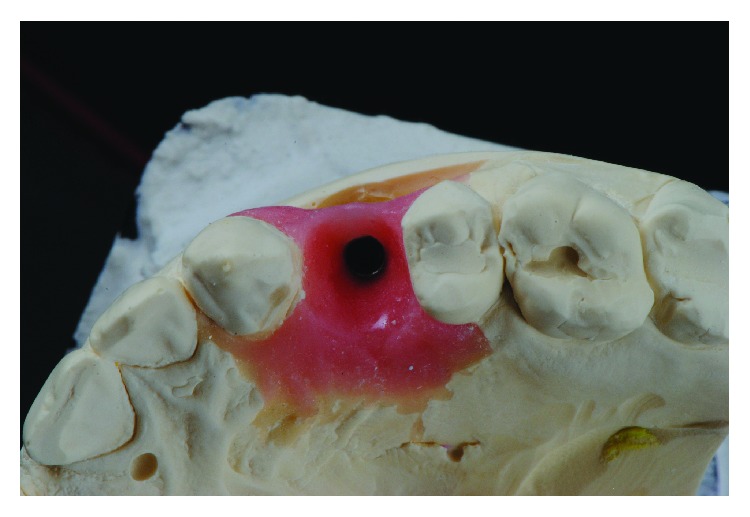
Working cast.

Even though the working cast reproduces the clinical situation faithfully (Figure 5), we proceeded with the rehabilitation using the CAD/CAM technology-customized zirconia (hybrid) for link abutment (FLEXIBASE®, Anthogyr) which offers advantages over prefabricated ones.

**Figure 6:**
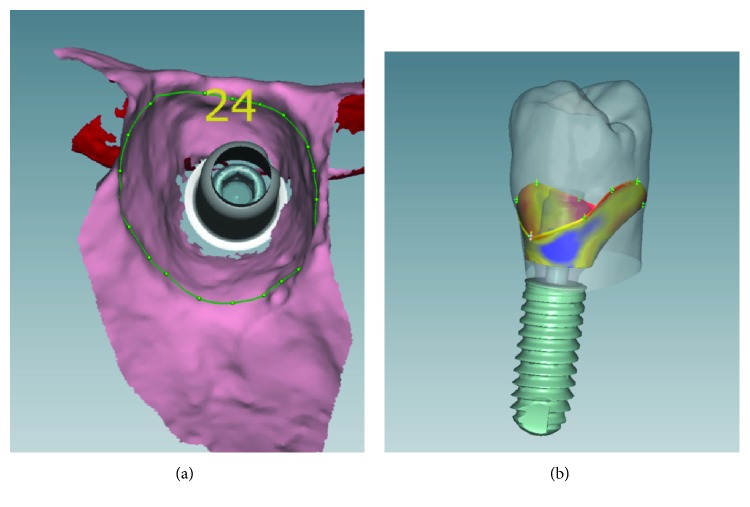
Gingival margin delimitation and abutment customization.

Figure 6(a) enables us to observe that through this technology, the gingival margin is delimitated in order to make the abutment emerge throughout the soft tissue as similar as a natural clinical crown (Figure 6(b)).

**Figure 7:**
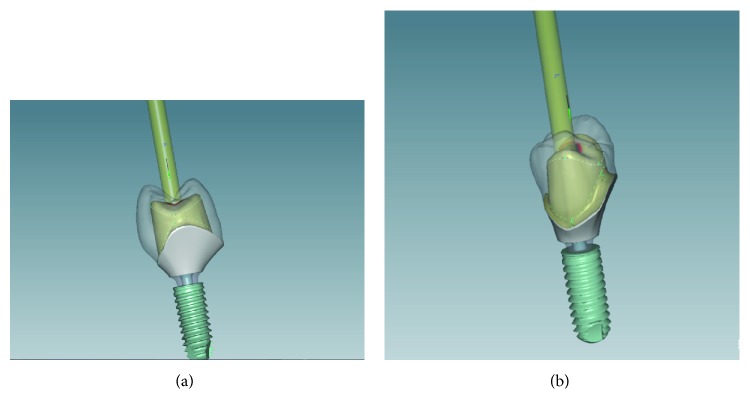
Divergent angle of implant trajectory and agglutination correction.

The zirconia project enables angular corrections in the trajectory position, in order to avoid or minimize differences between implant and crown position (Figures 7(a) and 7(b)).

**Figure 8:**
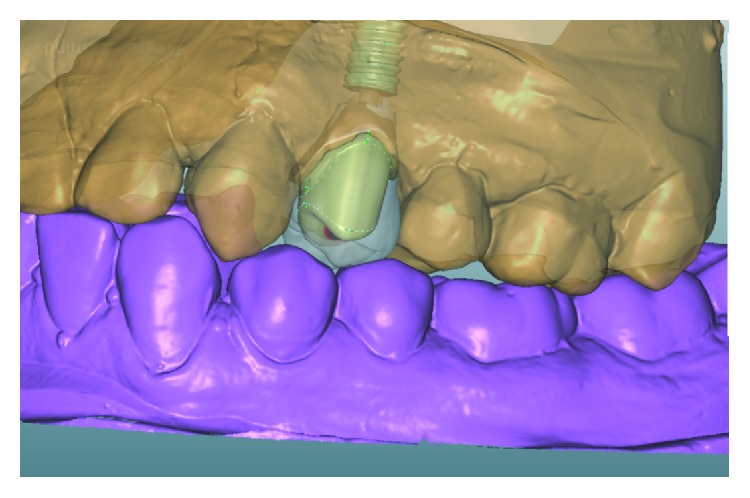
Adjustment of the proportion abutment/crown for ideal retention.

**Figure 9:**
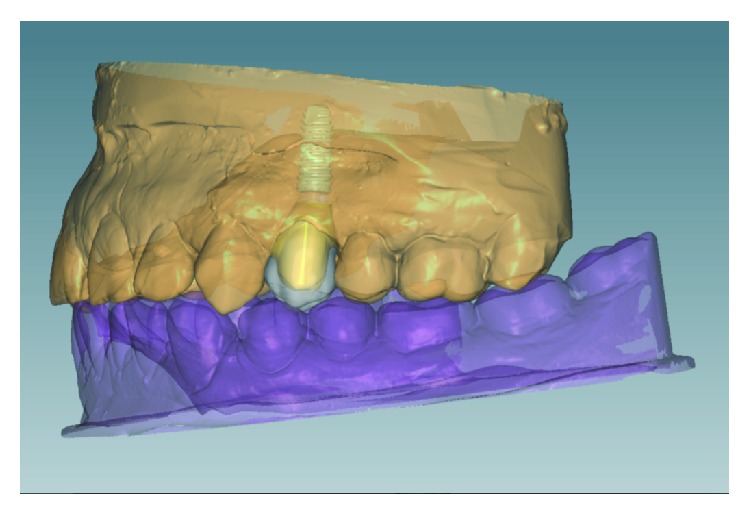
Interproximal adjustment and occlusal check.

Once the crown is designed, the outer part of the abutment is adjusted to create support and to provide retention which is achieved by planning an ideal proportion between the hybrid abutment and restorative crown, interocclusal space, and cementation line appropriated to the final restoration (Figures 8 and 9).

**Figure 10:**
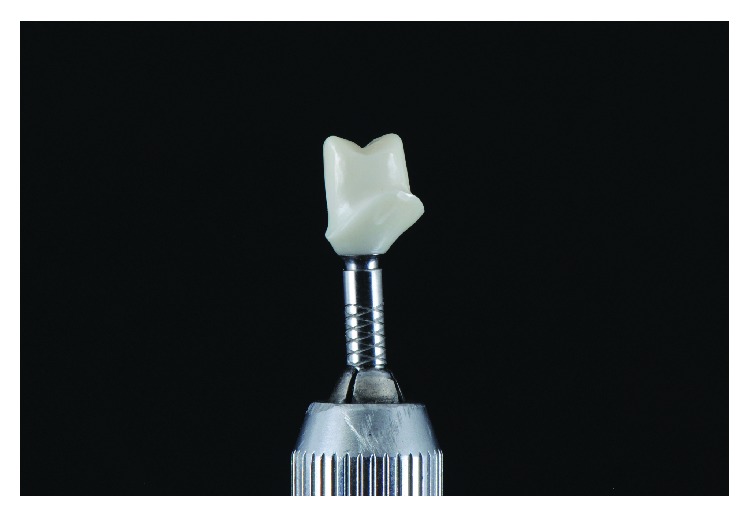
Zirconia of the hybrid abutment.

**Figure 11:**
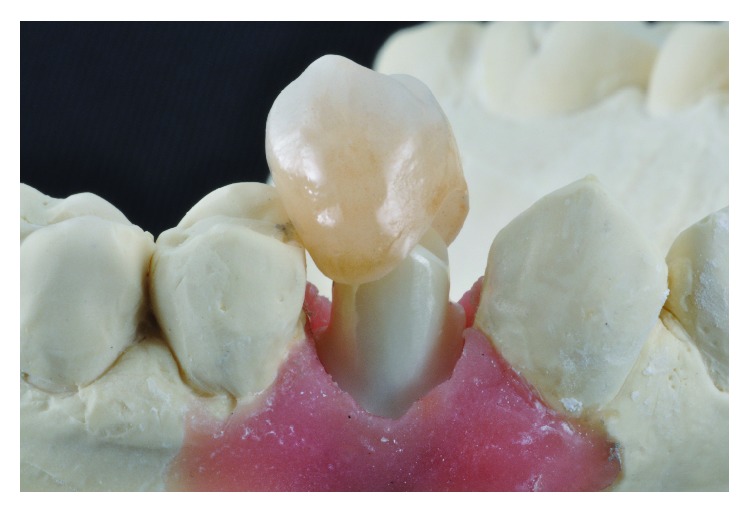
Zirconia of the hybrid abutment and E-max crown.

An E-max (ips-E-max, ivoclaire) pure crown final restoration was manufactured (Figures 10 and 11).

To cement the zirconia abutment in the link abutment, the flex base, the bonding surfaces of the titanium, and the zirconia ceramic were air-abraded with 50 mm aluminum oxide particles at 2.0 bars of pressure (0.25 MPa) for 20 seconds at a distance of 10 mm, after which they were cleaned in alcohol and then cemented using a resin luting (Relyx U200, 3M ESPE®) [18]. Excess resin was removed from the bonding margins before it became fully set and was light-cured per the manufacturer's recommendations.

**Figure 12:**
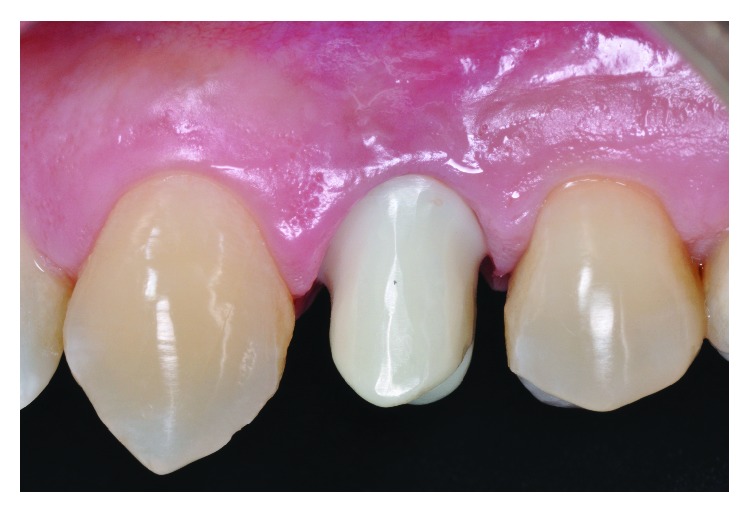
Abutment in position, manufactured in CAD/CAM technology.

The hybrid abutment was placed (Figure 12) with 25 N definitive torque, and the crown was cemented using a resin luting (Relyx U200, 3M ESPE).

**Figure 13:**
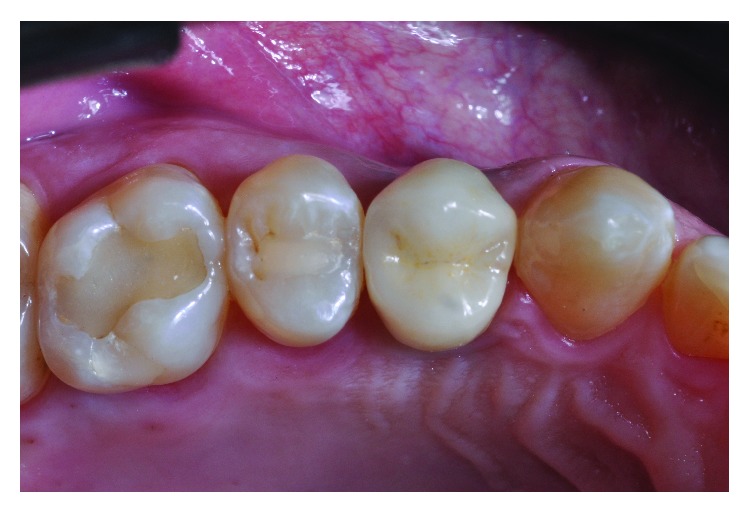
Cemented crown.

**Figure 14:**
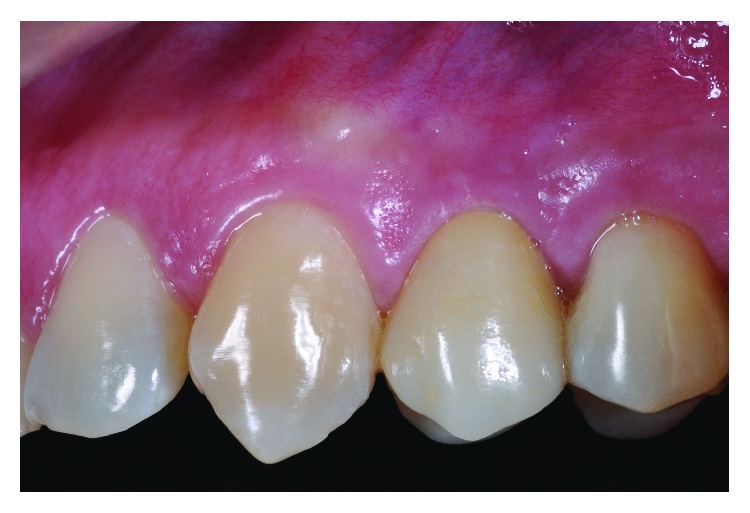
Crown as noticed by the color, shape, texture, and contour of the gingival tissue.

The clinical results (Figures 13 and 14), one month after prosthesis installation, prove the component adaptation placement and the quality in the contour of the gingival tissues. The successful aesthetic can be noticed by the smile harmony, color, texture, and natural brightness in comparison to the adjacent teeth.

## 3. Discussion

Dental implant treatment for dental element replacement considering the maintenance of gingival architecture and restoration has occurred for some years. However, by pursuing better aesthetics, hybrid abutments have surpassed metal abutments and provide a more natural appearance to the ceramic restorations.

CAD/CAM systems enabled the fabrication of the customized zirconia for link abutments that are individualized for both the anterior region and posterior teeth. Nowadays, through the CAD/CAM technology, hybrid abutment can be designed and manufactured ensuring mechanical characteristics of the materials [13, 19, 20].

CAD/CAM systems present several advantages, such as fast production, biocompatibility, aesthetics, and mechanical resistance with low fracture rate (because the blocks are industrially produced and have high homogeneity, without the need for refractory casts) [21, 22]. Furthermore, they enable excellent adaptation between margin restoration and soft tissues [23, 24].

There are several studies comparing metal and hybrid abutment characteristics [25]. Taking aesthetics into consideration, it was observed that hybrid abutments did not give grayish appearance to the gingival margin as noticed when metal abutments were used. This is a great advantage, especially to patients who have a high smile line and fine gingival genotype [3].

Studies have shown that the zirconia oxide presents mechanical resistance similar to titanium. This property combined with new automated techniques (CAD/CAM) made the use of hybrid abutment possible in rehabilitation both in the anterior region and in regions with higher masticatory load [13, 21, 24–28].

Material biocompatibility is very important for the longevity of implant-supported restorations. A great deal of studies observed that zirconia has presented low bacterial adherence, and hybrid abutments accumulate bacteria with lower pathogenic potential in relation to titanium abutments [6, 7].

Single hybrid abutments have aesthetics, mechanical resistance, and biocompatibility which enable metal components to be replaced in implanted supported prostheses. However, each case should be evaluated carefully as these components were recently introduced to the market and there are no long-term studies on their clinical use.
